# Atrophic changes in thyroid tumors are strong indicators of underlying *DICER1* mutations: a bi-institutional genotype–phenotype correlation study

**DOI:** 10.1007/s00428-024-03802-y

**Published:** 2024-04-19

**Authors:** Vincenzo Condello, James W. Roberts, Adam Stenman, Catharina Larsson, Kartik Viswanathan, C. Christofer Juhlin

**Affiliations:** 1 Department of Oncology-Pathology, Karolinska Institutet, Stockholm, Sweden; 2 Department of Pathology and Laboratory Medicine, Children’s Healthcare of Atlanta, Atlanta, GA USA; 3 Department of Breast, Endocrine Tumors and Sarcoma, Karolinska University Hospital, Stockholm, Sweden; 4 Department of Molecular Medicine and Surgery, Karolinska Institutet, Stockholm, Sweden; 5 Department of Pathology and Laboratory Medicine, Emory University School of Medicine, Atlanta, GA USA; 6 Winship Cancer Center, Decatur, GA USA; 7 Department of Pathology and Cancer Diagnostics, Karolinska University Hospital, Stockholm, Sweden

**Keywords:** *DICER1*, Somatic mutation, Atrophic changes, Macrofollicular pattern

## Abstract

Somatic and biallelic *DICER1* mutations are reported in subsets of thyroid tumors, supporting the role of this gene in thyroid tumor development. As recent studies have brought attention to macrofollicular patterns, atrophic changes, and papillary structures as being associated with *DICER1* mutations, we sought to explore these observations in a bi-institutional cohort. A total of 61 thyroid lesions (54 tumors and 7 cases of thyroid follicular nodular disease; TFND), including 26 *DICER1* mutated and 35 *DICER1* wildtype controls were subjected to histological re-investigation and clinical follow-up. *DICER1-*mutated lesions showed a statistically significant association with younger age at surgery (29.2 ± 12.5 versus 51.3 ± 18.8, *p* = 0.0001), a predominant macrofollicular growth pattern (20/26 mutated cases versus 18/35 wildtype; *p* = 0.01) and atrophic changes (20/26 mutated cases versus 2/35 wildtype; *p* = 0.0001). Similar results were obtained when excluding TFND cases. We also present clinical and histological triaging criteria for *DICER1* sequencing of thyroid lesions, which led to the identification of *DICER1* variants in 16 out of 26 cases (62%) when followed. Among these, 3 out of 12 cases with available data were found to carry a constitutional *DICER1* mutation. This observation suggests that the majority of *DICER1* mutations are somatic—however implies that sequencing of constitutional tissues could be clinically motivated. We conclude that *DICER1* mutations are amassed in younger patients with macrofollicular-patterned tumors and, most strikingly, atrophic changes. Given the rate of constitutional involvement, our findings could be of clinical value, allowing the pathologist to triage cases for genetic testing based on histological findings.

## Introduction

Hereditary cancer syndromes account for approximately 10% of all cancers [1]. The proportion of patients with thyroid tumors exhibiting a monogenic, syndromic form of the disease is very low when considering tumors from follicular-derived cells [2]. The most established entities in this context are the McCune-Albright and PTEN syndromes, where a certain proportion of patients develops follicular thyroid tumors [3–6].

The discovery of two separate syndromes caused by mutations in genes controlling the maturation of micro-RNA (*DICER1* and *DGCR8*) has been particularly interesting from a thyroid point of view, as these patients develop thyroid follicular nodular disease (TFND) and thyroid tumors [7, 8]. The DICER1 syndrome is an autosomal dominant disorder that places carriers at risk of developing multiple tumors and tumor-like conditions in the thyroid gland, lungs, gonads, and soft tissues [9]. The prevalence of this syndrome is relatively unknown but is believed to increase as general knowledge grows, leading to an intensification of clinical screening efforts [10]. The majority of patients with this syndrome carries an inactivating mutation in the *DICER1* gene, typically a prematurely truncated variant upstream of the functionally important RNAse III domain [11, 12]. However, missense mutations, in-frame deletions, and intronic variants affecting splicing have also been demonstrated [13–15]. Tumors in the context of this syndrome often exhibit a two-hit inactivation, where a somatic mutation is recruited on the second allele—an alteration frequently located in the functionally important domain that controls the processing of micro-RNA [16, 17]. For this reason, the gene is considered to be a tumor suppressor, which has also been demonstrated functionally in genetically engineered mice [18]. In contrast to constitutional mutations, somatic *DICER1* mutations typically occur at specific positions that are important for metal-ion binding such as the hotspot amino acids 1705, 1709, 1809, 1810, and 1813 [17].

Concerning sporadic thyroid tumors, it is known that mutations in this gene occur in a subset of TFND, follicular thyroid adenoma (FTA), non-invasive follicular thyroid neoplasm with papillary-like nuclear features (NIFTP), follicular thyroid carcinomas (FTC), and the invasive encapsulated follicular variant of papillary thyroid cancer (IEFVPTC) [19–23]. Mutations also manifest in a subset of high-grade thyroid tumors in pediatric patients, indicating a broad spectrum [24]. Recent work demonstrates a frequency of less than 5% for these mutations in various follicle cell-derived tumors [25]. Most of the *DICER1* mutations are somatic and typically do not occur concurrently with other driver mutations. This suggests that the gene has the potential to independently drive tumor development.

Interestingly, specific histologic attributes have been associated with these mutations. For instance, macrofollicular structures, “abortive areas,” and focal papillary growth have been associated with the presence of a *DICER1* mutation [26, 27].

In this study, we aimed to delineate these phenomena in a bi-institutional cohort of thyroid lesions with *DICER1* mutation, to potentially confirm the hypothesis that the mutation gives rise to specific histological attributes observable under the microscope. Furthermore, by employing a set of clinical and histologic inclusion criteria in clinical practice, we have attempted to evaluate how these factors might contribute to an increased proportion of mutation-positive findings in the clinical setting.

## Material and methods

### Detailed case description

A total of 61 thyroid lesions with available *DICER1* genotype (54 tumors and 7 TFND) were studied, including 26 *DICER1-*mutated cases and 35 wildtype controls. The summarized cohort information is shown in Table 1. Cases were collected from two independent institutions: 26 lesions from 26 patients diagnosed at the Karolinska University Hospital, Stockholm, Sweden, during 2021–2023 (Karolinska cohort), and 35 lesions from 33 patients diagnosed at Emory University Hospital, Atlanta, and Children’s Healthcare of Atlanta, both in Georgia, USA, collected during 2018–2023 (Emory/CHOA cohort). All Karolinska cases are previously unpublished, as are the majority of the Emory/CHOA cohort cases [27]. Ethical permission was granted through the Swedish Ethical Review Authority (approvals #2015.959–31 and # 2020–00281) and the Institutional Review Board (IRB#00004304), that includes both Emory University and Children’s Healthcare of Atlanta.

**Table 1:** Clinicopathological characteristics of the 61 thyroid nodules with available *DICER1* genotype

	Benign, *n* = 22 (36.1%)		Low risk, *n* = 6 (9.8%)		Malignant, *n* = 33 (54.1%)				
	TFND *n* = 7	FTA *n* = 15	NIFTP *n* = 2	FT-UMP *n* = 4	FTC *n* = 23	OTC *n* = 3	DHGTC *n* = 5	PDTC *n* = 2	All samples *n* = 61
Sex									
Female	6	14	2	2	21	2	3	1	51
Male	1	1	0	2	2	1	2	1	10
Age (years) [mean ± SD] Median (range)	37.7 ± 13.7 41 (15–52)	39.7 ± 18.3 37 (19–78)	23.5 ± 2.1 23.5 (22–25)	69.0 ± 13.5 74 (49–79)	40.8 ± 19.1 36 (13–83)	60.3 ± 12.4 67 (46–68)	38.4 ± 26.1 25 (17–78)	31.0 ± 22.6 31 (15–47)	41.9 ± 19.6 41 (13–83)
Nodule size (cm) [mean ± SD] Median (range)	4.4 ± 3.8 3.1 (1.8–12)	3.2 ± 1.6 3 (1.2–6.3)	4.9 ± 4.1 4.9 (2.1–7.8)	4.6 ± 2.1 4.9 (2–6.7)	3.1 ± 1.2 2.8 (1.2–5.2)	4.2 ± 0.5 3.9 (3.9–4.8)	3.8 ± 1.8 2.8 (2.3–6.5)	2.5 ± 0 2.5 (2.5)	3.5 ± 1.8 3 (1.2–7.8)
Tumor invasiveness									
Minimally invasive	-	-	-	-	20	1	-	-	21
Encapsulated angioinvasive	-	-	-	-	2	2	-	-	4
Widely invasive	-	-	-	-	1	0	-	-	1

*TFND*, thyroid follicular nodular disease; *FTA*, follicular thyroid adenoma; *NIFTP*, non-invasive follicular thyroid neoplasm with papillary-like nuclear features; FT-UMP, follicular tumor of uncertain malignant potential; *FTC*, follicular thyroid carcinoma; *OTC*, oncocytic thyroid carcinoma; *DHGTC*, differentiated high-grade thyroid carcinoma; *PDTC*, poorly differentiated thyroid carcinoma; *SD*, standard deviation

**Fig. 1:**
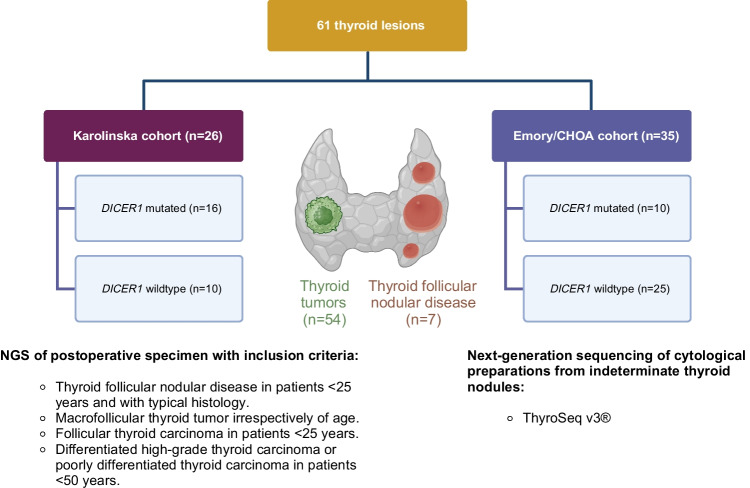
Schematic overview of the study design with inclusion criteria for genetic testing. Created using BioRender.com

At Karolinska, routine preoperative assessments of thyroid nodules do not typically involve next-generation sequencing (NGS) or direct sequencing of thyroid-related genes, except in rare cases. Hence, thyroid nodules from Karolinska that underwent *DICER1* sequencing by NGS were chosen based on clinical and histological screening, requiring the fulfillment of at least one among several specific criteria, and thereafter sequenced using DNA extracted from postoperative, formalin-fixated paraffin-embedded (FFPE) specimen (Fig. 1). The inclusion criteria for *DICER1* sequencing at Karolinska were (1) TFND in patients < 25 years and with typical histology (multiple adenomatoid nodules with papillae), (2) histological diagnosis of a macrofollicular thyroid tumor (FTA, FTC, papillary thyroid carcinoma; PTC) irrespectively of age, (3) conventional FTC in patients < 25 years, and (4) differentiated high-grade thyroid carcinoma (DHGTC) or poorly differentiated thyroid carcinoma (PDTC) in patients < 50 years. Meaning, all cases from Karolinska in this study have fulfilled one of the abovementioned criteria, irrespective of whether they harbored a *DICER1* gene mutation or not.

For the Emory/CHOA cohort, the respective pathology archives were queried for indeterminate cytology cases that underwent Thyroseq v3 testing and found to have a *DICER1* mutation. Corresponding surgical resection samples, when available, were collected and reviewed. In addition to the *DICER1*-mutated cases, a separate group of resected *DICER1* wildtype, *RAS*-like mutant thyroid lesions were selected to serve as control cases.

Baseline clinical and histopathological data were gathered for the entire study cohort by reviewing patient charts and pathology reports. Additionally, a meticulous histological review was conducted, focusing on specific histological attributes. Specifically, atrophic changes, previously referred to as “abortive changes” [26], were characterized by the presence of multiple areas in which the tumor tissue appeared atrophic and palely stained compared to the surrounding tissue, with ghost-cell-like areas exhibiting clear cut demarcation compared to the unaffected, adjacent tumor tissues. Usually, these areas exhibited thickening of the interstitial stroma and a reduction of viable cells. These changes were frequently observed in tumor regions adjacent to the capsule. It is important to note that our definition of atrophic changes excludes features such as associated fibrosis, inflammation, and cellular debris. This exclusion serves to safely eliminate potential artifacts stemming from post-fine needle aspiration biopsy.

### *DICER1* sequencing of thyroid lesions

For Karolinska cases, DNA was extracted from FFPE and sequenced using the Oncomine Childhood Cancer NGS platform (Thermo-Fisher, Waltham, MA, USA) used in clinical routine at the Department of Pathology and Cancer Diagnostics. This platform includes the entire coding sequence and intron–exon boundaries of *DICER1*, in addition to a number of other cancer-related genes and fusions, e.g., *BRAF*, *NRAS*, *HRAS*, and *KRAS* to name a few. The report obtained includes the *DICER1* genotype and allele frequency of each mutation found. All samples were confirmed as containing high tumor purity via histological investigation of a representative section from the DNA extraction process.

For the Emory/CHOA, the indeterminate cases on cytology underwent Thyroseq v3 testing as described previously [28], which identified a *DICER1* or a *RAS*-like alteration (e.g., *RAS*, *EIF1AX*).

### Constitutional *DICER1* mutation detection

A subset of Karolinska cases with identified *DICER1* mutation in the thyroid lesion underwent scrutiny for constitutional involvement through the Clinical Genetics Department, utilizing an NGS platform employed in routine clinical practice.

### Statistical analyses

Fisher’s exact test and Mann–Whitney-*U* test were used to compare clinicopathological features between *DICER1*-mutated and wildtype cases, and a *p*-value < 0.05 was considered statistically significant. Statistical computations were performed using GraphPad Prism 10.1.1 (GraphPad Software, San Diego, California USA).

## Results

### *DICER1* genotype and correlations to clinical and histological variables

Out of the 61 thyroid lesions (54 tumors and 7 cases of TFND), 26 lesions harbored *DICER1* mutations and the remaining 35 were wildtype. Genetic data, clinical, and histological features for all *DICER1*-mutated lesions are detailed in Table 2. Totally, 25 of the 26 *DICER1*-mutated thyroid lesions exhibited a hotspot mutation, which was shown to be somatic in all 14 for which constitutional DNA was sequenced. Another thyroid lesion exhibited a nonsense mutation that was shown to be constitutional.

**Table 2:** Clinical and genetic characteristics of all *DICER1*-mutated cases

Case ID	Diagnosis	Sex	Age, years	Nodule size, cm	Architectural pattern	Papillary formations	Atrophic changes	*DICER1* hotspot mutation in thyroid lesions (AF%)	Constitutional *DICER1* mutation (AF%)	Other mutations apart from *DICER1* in thyroid lesions (AF%)
EC 1	FTA	F	19	5.5	Macrofollicular	YES	NO	c.5126 A > G p.D1709G (32%)	NP	None
EC 2	NIFTP	F	22	7.8	Macrofollicular	YES*	NO	c.5126 A > G p.D1709G (30%)	NP	None
EC 3	MIFTC	F	36	2.5	Macrofollicular	YES	YES	c.5439 G > T p.E1813D (63%)	NP	None
EC 4^§^	PDTC	F	15	2.5	Microfollicular	NO	YES	c.5127 T > G p.D1709E (27%)	c.5464 G > A p.D1822N (VUS)	None
EC 5^§^	FTA	F	15	1.2	Macrofollicular and solid	NO	YES	c.5437 G > A p.E1813K (15%)	c.5464 G > A p.D1822N (VUS)	None
EC 6	FTA	F	49	1.5	Microfollicular	NO	YES	c.5126 A > G p.D1709G (37%)	NP	None
EC 7	FTA	F	55	2	Macrofollicular	NO	YES	c.5126 A > G p.D1709G (43%)	NP	None
EC 8	DHGTC	M	25	2.8	Macrofollicular	YES	YES	c.5429 A > T p.E1810V (60%)	NP	None
EC 9	MIFTC	F	17	2	Macrofollicular	YES	YES	c.5126 A > G p.D1709G (41%)	WT	None
EC 10	MIFTC	F	22	3.2	Microfollicular	YES	YES	c.5429 A > T p.E1810V (51%)	NP	None
KI 1	TFND	F	15	12	Macrofollicular	YES	YES	NO	c.4154_4155ins p.Y13* (48%)	None
KI 2	MIFTC	F	13	1.2	Macrofollicular	YES	YES	c.5126 A > G p.D1709G (20%)	c.2830 C > T p.R944* (50%)	*FGFR4* p.T179A (51%)
KI 3	MIFTC	F	29	5	Macrofollicular	YES	YES	c.5437 G > C p.E1813Q (39%)	c.2908 G > T p.E790* (40%)	None
KI 4	MIFTC	F	33	1.5	Macrofollicular	YES	YES	c.5439 G > T p.E1813D (39%)	WT	None
KI 5	MIFTC	F	44	2.8	Macrofollicular	YES	YES	c.5428 G > T p.E1810Y (40%)	WT	None
KI 6	DHGTC	F	20	6.5	Microfollicular	NO	NO	c.5437 G > A p.E1813K (27%)	WT	None
KI 7	MIFTC	F	27	2.5	Macrofollicular	YES	YES	c.5126 A > G p.D1709G (11%)	WT	None
KI 8	FTA	F	31	4	Macrofollicular	YES	YES	c.5428 G > T p.E1810Y (42%)	WT	None
KI 9	FTA	F	51	4.2	Macrofollicular	NO	YES	c.5437 G > C p.E1813Q (34%)	WT	None
KI 10	DHGTC	F	17	2.5	Microfollicular	NO	YES	c.5437 G > A p.E1813K (57%)	NP	None
KI 11	FTA	F	30	3.5	Macrofollicular	NO	YES	c.5437 G > A p.E1813K (43%)	WT	None
KI 12	PDTC	M	47	2.5	Trabecular, solid	NO	NO	c.5126 A > T p.D1709V (69%)	WT	None
KI 13	MIFTC	F	27	4.5	Macrofollicular	YES	YES	c.5437 G > C p.E1813Q (25%)	WT	*SMARCB1* p.F25L (46%)
KI 14	TFND	F	41	4.5	Macrofollicular	YES	NO	c.5126 A > G p.D1709G (39%)	NP	None
KI 15	MIFTC	F	38	2.8	Macrofollicular	YES	YES	c.5126 A > G p.D1709G (17%)	NP	*PTPN11* p.A72T (11%)
KI 16	TFND	F	23	3.2	Macrofollicular	YES	NO	c.5126 A > G p.D1709G (37%)	NP	*KRAS* p.Q61L (24%)

EC, Emory/CHOA cohort; *KI*, Karolinska Institutet cohort; *TFND*, thyroid follicular nodular disease; *FTA*, follicular thyroid adenoma; *NIFTP*, non-invasive follicular thyroid neoplasm with papillary-like nuclear features; *MIFTC*, minimally invasive follicular thyroid carcinoma; *DHGTC*, differentiated high-grade thyroid carcinoma; *PDTC*, poorly differentiated thyroid carcinoma; *NP*, not performed; *AF*, allele frequency; *VUS*, variant of uncertain significance; §: lesions from the same patient. *Papillary formations were noted in < 1% of the lesion, in agreement with the diagnostic criteria for NIFTP

In brief, the 26 *DICER1*-mutated thyroid lesions include 3 TFND cases, 8 benign/low-risk lesions (7 FTA and 1 NIFTP), 10 well-differentiated thyroid carcinomas (10 FTC), and 5 high-grade thyroid carcinomas (3 DHGTC and 2 PDTC). The 35 *DICER1* wildtype thyroid lesions consist of 4 TFND cases, 13 benign/low-risk lesions (8 FTA, 4 follicular thyroid tumors of uncertain malignant potential; FT-UMP, 1 NIFTP), 16 well-differentiated thyroid carcinomas (13 FTC, 3 oncocytic thyroid carcinoma; OTC), and 2 high-grade thyroid carcinomas (both DHGTC).

**Fig. 2:**
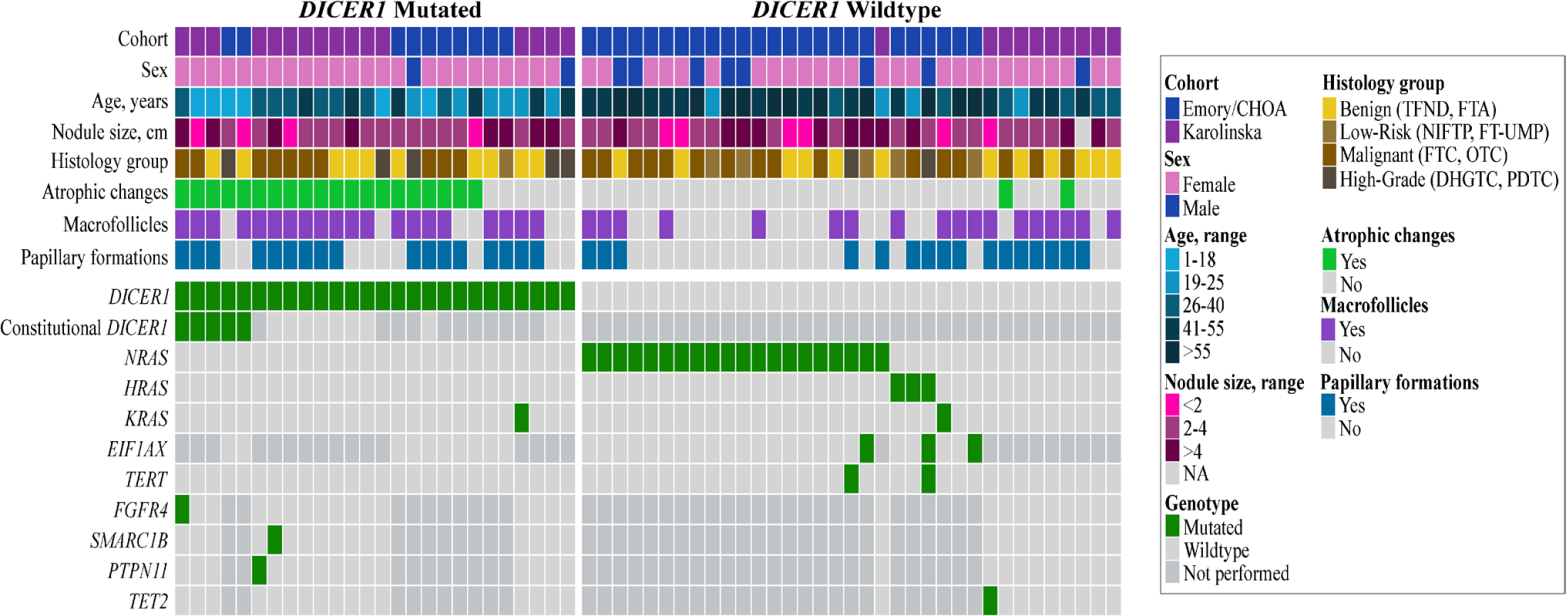
Oncoplot with associated clinical and histological features of the 61 thyroid lesions included in the study

**Fig. 3:**
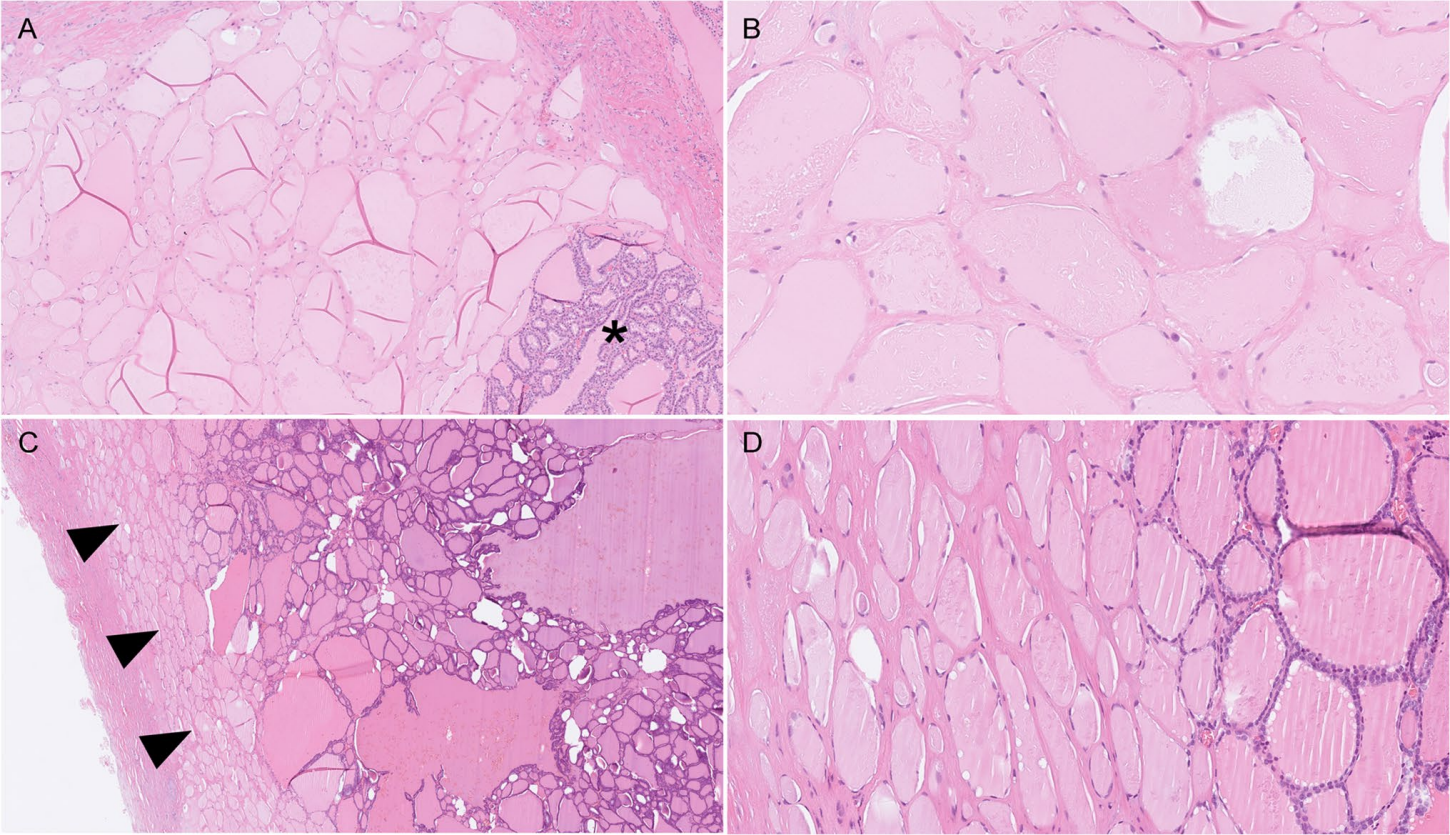
**A** Atrophic changes in case KI 2, a minimally invasive follicular thyroid carcinoma (MIFTC) with macrofollicular architecture in a patient with constitutional *DICER1* mutation. Widespread areas with palely stained tumor cells (termed “atrophic changes”) were noted adjacent to the non-affected tumor cells (asterisk). The absence of cellular debris and inflammation argues against necrosis, and there are no apoptotic bodies. Magnification × 100. **B** High-magnification (× 400) photomicrograph of the same case, highlighting the atrophic changes. These features are sensitive and highly specific for *DICER1* mutations in follicular-patterned thyroid tumors. **C** Case KI 8 at × 40 magnification, a macrofollicular variant follicular thyroid adenoma with somatic *DICER1* mutation. Note the brisk transition between atrophic changes (arrowheads) and unaffected tumor tissue. **D** High-power magnification of the same case

**Fig. 4:**
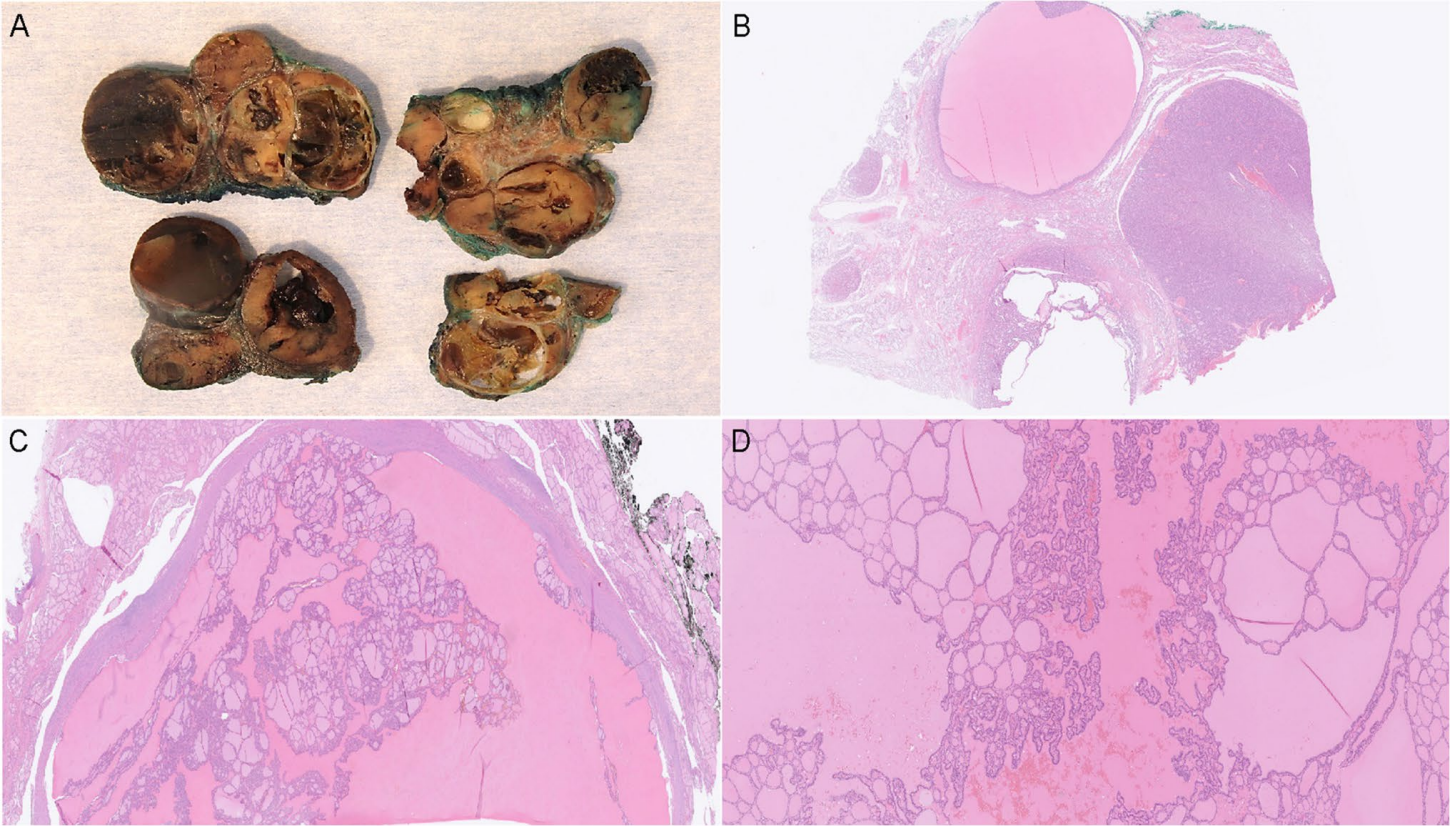
Main histological attributes of KI 1 case, a thyroid follicular nodular disease (TFND) in a young female patient with germline *DICER1* mutation. **A** Macroscopic image. Note the heterogeneous appearance with multiple adenomatoid-like nodules visible at grossing. **B** Hematoxylin and eosin (H&E) staining of the same case, displaying colloid nodules mixed with microfollicular-patterned adenomatous areas. **C–D** Areas with prominent papillary formations are evident. Nuclear atypia is absent

The results are summarized in Fig. 2. *DICER1-*mutated cases displayed a younger age at surgery (29.2 ± 12.5 vs. 51.3 ± 18.8, *p* ≤ 0.0001), a predominant macrofollicular growth pattern (20/26 mutated cases vs. 18/35 wildtype; *p* = 0.01) and atrophic changes (20/26 mutated cases vs*.* 2/35 wildtype; *p* ≤ 0.0001) (Table 3; Fig. 3). Papillary formations were not statistically significantly overrepresented among *DICER1*-mutated lesions but were a recurrent feature of TFND cases with *DICER1* mutations (Table 3; Fig. 4). Notably, atrophic changes were highly sensitive and specific for the detection of *DICER1* mutations. In the thyroid tumors studied, atrophic changes were noted in 20 out of 26 *DICER1*-mutated cases (77%), compared to 2 out of 35 *DICER1* wild-type cases (5%). Thus, the sensitivity and specificity of atrophic changes as a harbinger of *DICER1* mutations would reach 77% and 95% respectively. Atrophic changes were observed both in cases with somatic as well as constitutional variants, as illustrated in Fig. 3, and there was no apparent association with mutational status in this regard.

**Table 3:** Summary of the clinicopathological features of all the *DICER1*-mutated versus wildtype cases

	*DICER1* mutants (*n* = 26)	*DICER1* wildtype (*n* = 35)	*p*-value
Sex			
*Female*	24	27	0.16
*Male*	2	8	
Age, years (mean ± SD)	29.2 ± 12.5	51.3 ± 18.8	0.0001*
Nodule size, cm (mean ± SD)	3.6 ± 2.3	3.4 ± 1.4	0.82
Architectural pattern			
*Macrofollicular*	20	18	0.01*
*Microfollicular* *Other*	4 2	17 0	
Papillary growth^§^			
*Yes*	17	16	0.19
*No*	9	19	
Atrophic changes			
*Yes*	20	2	0.0001*
*No*	6	33	

Fisher’s exact test and Mann–Whitney multiple comparison test were used. *p*-values < 0.05 were considered significant. *Significant *p*-value. §Findings of true papillary structures irrespectively of the proportion of tumor volume

### Constitutional *DICER1* sequencing outcome

In the Karolinska cohort, *DICER1* mutations were found in 16 out of 26 cases (62%) fulfilling the inclusion criteria. In cases with available germline data (*n* = 12), 3 cases (25%) were found to carry constitutional *DICER1* nonsense mutations: in one patient with TFND and two patients with minimally invasive FTC (MIFTC) with macrofollicular growth. These patients were females aged 13, 15, and 29 at surgery. Two of these patients exhibited a family history of TFND, and the grandmother of an additional patient was previously diagnosed with thyroid carcinoma. In the remaining 9 cases, no constitutional *DICER1* mutation was identified.

In the Emory/CHOA cohort, two patients with *DICER1*-mutated thyroid lesions identified on Thyroseq v3 underwent constitutional DNA testing. One female patient aged 15 with a PDTC and a separate FTA exhibited a constitutional *DICER1* missense variant of uncertain significance (VUS) (Table 2). The second patient tested, aged 17 and female, had a MIFTC on surgery. This patient did not have a constitutional *DICER1* alteration and no significant family history.

**Fig. 5:**
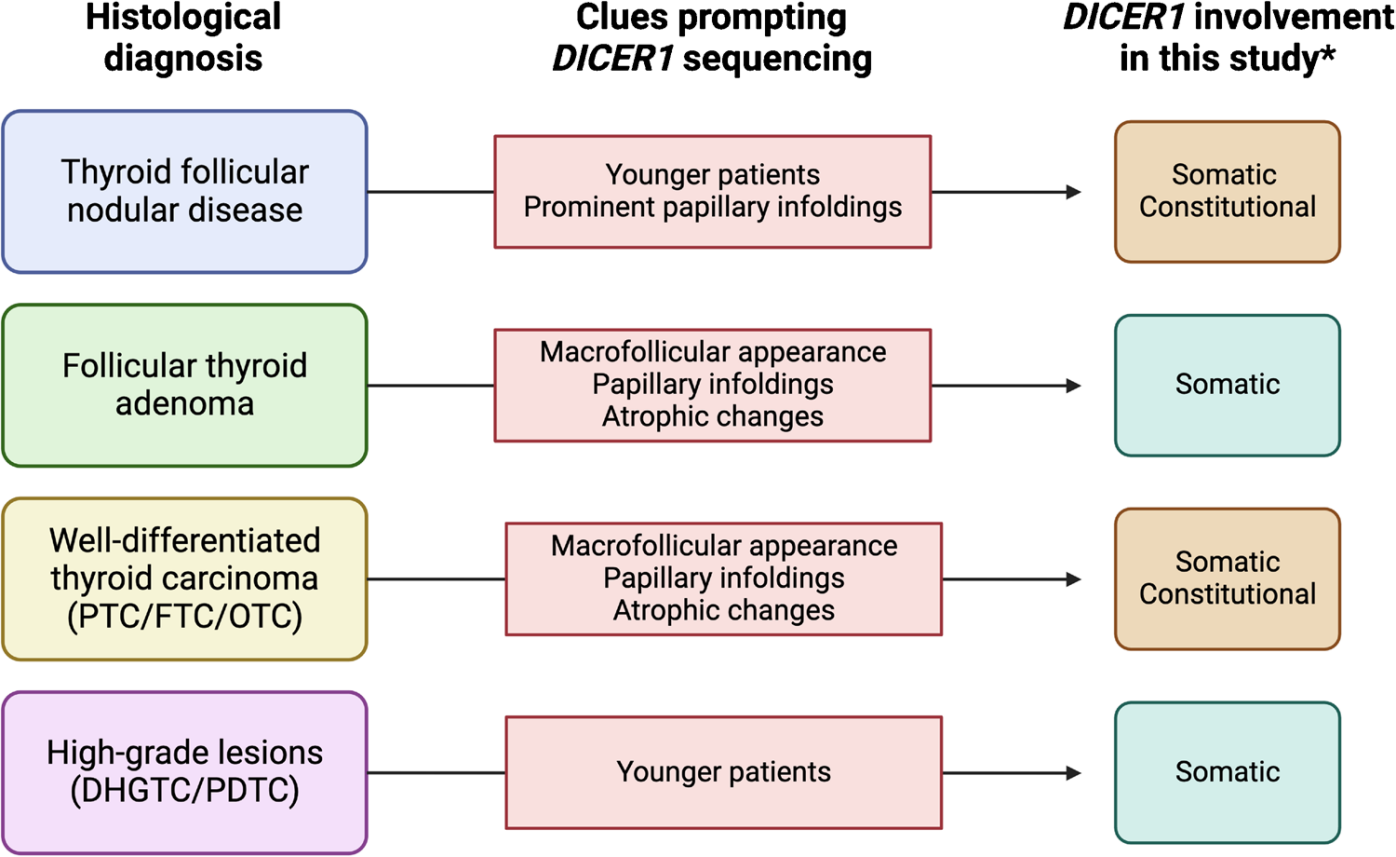
*DICER1* screening in thyroid lesions using clinical and histological harbingers: a suggested algorithm for centers that do not perform universal genetic testing in thyroid nodules. *Data from the current study with a limited number of Karolinska patients undergoing germline sequencing. PTC, papillary thyroid carcinoma; FTC, follicular thyroid carcinoma; OTC, oncocytic thyroid carcinoma; DHGTC, differentiated high-grade thyroid carcinoma; PDTC, poorly differentiated thyroid carcinoma. Created using BioRender.com

Overall, based on these results, we suggest an algorithm, summarized in Fig. 5, that could be used in centers that routinely do not perform universal genetic testing in thyroid nodules, to better predict *DICER1* mutations using various clinical and histological criteria.

## Discussion

Characterized for the first time in 2009, the DICER1 syndrome is a multi-tumor condition caused by germline pathogenetic variants in the *DICER1* gene and associated with different neoplastic lesions affecting the lungs, thyroid gland, ovaries, and kidney [7]. For the syndrome to occur, typically, a loss-of-function variant, resulting in the inactivation of the affected *DICER1* allele, and a second “hotspot” pathogenetic mutation affecting the RNase IIIb domain of *DICER1* are required [12].

Patients with DICER1 syndrome, typically in pediatric or adolescent age, face an increased risk of developing a variety of thyroid lesions, spanning from TFND to malignant thyroid tumors [29–31]. Even though this syndrome is important to recognize, it should be stressed that somatic *DICER1* aberrations in thyroid nodules are likely to be more prevalent than germline mutations [17]. Given the rather widespread occurrence of these somatic mutations in thyroid nodules, there is a significant clinical need to ascertain the clinical relevance and extent of these alterations, as well as whether the finding of these alterations would require germline testing to rule out syndromic disease [19, 21, 32, 33]. Moreover, as the detection rate of *DICER1* mutations in thyroid tumors will rise as more pathology centers rely on next-generation sequencing, identifying potential genotype–phenotype correlations is crucial, especially for centers that require specific conditions for case submission for sequencing.

In this study, we gathered molecular, histopathological, and clinical data from thyroid tumors at two distinct institutions (European and North American) to examine whether our initial observations regarding a genotype–phenotype correlation remain consistent in larger cohorts obtained from diverse centers. Our investigation affirms that specific histological features, such as macrofollicular growth and atrophic changes distinctly correlate with the presence of *DICER1* mutation in follicular-patterned thyroid tumors, corroborating the previously assumed association between macrofollicular variants of follicular thyroid tumors and inactivating somatic *DICER1* mutations reported in previous studies [26, 27, 34, 35]

Interestingly, the identification of atrophic areas seems to be strongly associated with this genetic aberrancy, as only 2 out of 35 *DICER1* wildtype cases with *RAS* mutations exhibited such areas. It is not known if atrophic changes are unique to follicular cell-derived thyroid tumors or if they are also visualized in unrelated thyroid tumors with *DICER1* mutations, for example, thyroblastoma [36, 37]. Upon reviewing the current literature, we did not observe such an association. However, future research investigating the potential role of atrophic changes in this tumor type would indeed be of interest in order to increase our understanding of this phenomenon.

Moreover, while atrophic changes have not yet been described on the cytological level, there are reports suggesting that macrofollicular structures and papillary excrescences may also be detected in fine needle aspirates of *DICER1*-mutated thyroid nodules [38]. Further studies in cytological preparations could possibly help delineate important clues already at the preoperative level. The specific mechanism underlying the atrophic changes is not known, but one might speculate that the aberrant micro-RNA expression patterns conferred by the *DICER1* mutations potentially instigate an atrophic state. Indeed, several micro-RNAs have been associated with thyrocyte maturation and differentiation [39]. Moreover, DICER1 deficiency in the retinal pigment epithelium has been associated with oxidative stress and geographic atrophy, and *DICER1* knock-out mice develop prostate atrophy [40, 41].

The *DICER1-*mutated cohort from Karolinska was assembled via NGS analysis, specifically targeting cases that exhibited specific epidemiologic and/or histologic criteria. By screening only those lesions meeting specific criteria, the percentage of positive cases—indicating patients with the aforementioned mutation—comprises > 50% of the total material. This should be compared to observations made in a large, unselected material of > 400 TFND and follicular-patterned tumors in which the *DICER1* hotspot mutational frequency was approximately 3% [25]. This underscores our demonstration that employing specific criteria can substantially elevate the proportion of patients with detected *DICER1* mutation in clinical practice.

In the present study, not all patients underwent analysis for mutations in constitutional tissue. Additionally, prior research indicates that most of these mutations are somatic [19, 20]. The critical question, therefore, revolves around the significance of detecting such a genetic aberration in clinical routine. Presumably, this approach allows pathologists to guide cases to surgeons for further clinical genetic management. Although the majority of patients may test negative for constitutional mutations, identifying individuals with syndromic forms of the disease holds substantial clinical value, justifying meticulous genetic mapping. Nevertheless, firm conclusions in this regard necessitate larger studies with available constitutional data.

To summarize, based on morphological and histological characteristics, we suggest an algorithm that could be used to predict *DICER1* mutations in follicular-patterned thyroid tumors a priori. In particular, specific features such as macrofollicular growth and atrophic changes appear to be strongly coupled to *DICER1* mutations in follicular-pattern thyroid tumors. Potentially, these histological attributes, together with young age, may display a specific and unique combination that could be of clinical interest, allowing pathologists to categorize cases for genetic testing based on histological findings.

## Data Availability

All data generated or analyzed during this study are included in this published article.
